# Cortical and Striatal Functional Connectivity in Juvenile-Onset Huntington’s Disease

**DOI:** 10.3390/brainsci15060663

**Published:** 2025-06-19

**Authors:** Amy Barry, Peg C. Nopoulos

**Affiliations:** 1 Interdisciplinary Graduate Program in Neuroscience, University of Iowa, Iowa City, IA 52242, USA; amy-barry@uiowa.edu; 2 Department of Psychology and Brain Sciences, University of Iowa, Iowa City, IA 52242, USA; 3 Department of Psychiatry, Carver College of Medicine, University of Iowa, Iowa City, IA 52242, USA; 4 Department of Neurology, Carver College of Medicine, University of Iowa, Iowa City, IA 52242, USA; 5 Department of Pediatrics, Carver College of Medicine, University of Iowa, Iowa City, IA 52242, USA

**Keywords:** juvenile-onset Huntington’s disease, striatal hypoconnectivity, resting-state functional connectivity, somatomotor network, antagonistic pleiotropy

## Abstract

Background: Huntington’s disease (HD) is a neurodegenerative disorder caused by a CAG repeat expansion in the HTT gene, with a rare juvenile-onset form (JoHD) marked by early, rigid motor symptoms. This study examined cortical and subcortical resting-state connectivity in JoHD, hypothesizing preserved cortical networks but altered striatal connectivity, in line with early subcortical atrophy despite relatively spared cortical volume. Methods: Participants included children and young adults with clinician-confirmed Juvenile-Onset Huntington’s Disease (JoHD; *n* = 19) and gene-non-expanded (GNE) controls *(n* = 64), both drawn from longitudinal studies at the University of Iowa. Resting-state functional MRI scans were analyzed to assess canonical cortical network and striatal connectivity, and linear mixed-effects models tested group differences and associations with motor, cognitive, and clinical outcomes. Results: JoHD participants showed reduced connectivity within the left somatomotor network and striatal circuits, despite largely typical cortical network connectivity. Striatal connectivity was associated with disease burden and cognitive ability, while left somatomotor connectivity was unrelated to clinical outcomes. Conclusions: These findings support the hypothesis of antagonistic pleiotropy in JoHD, where early neural advantages—such as relatively preserved or possibly enhanced cortical function—may contribute to later striatal vulnerability and degeneration. The observed left-lateralized somatomotor hypoconnectivity aligns with prior volumetric and gene expression research, highlighting the role of excitotoxic glutamatergic input and the selective vulnerability of high-functioning circuits in disease progression.

## 1. Introduction

Huntington’s disease is an autosomal dominant neurogenerative disorder that occurs when the huntingtin gene (HTT) contains an excessive expansion of the CAG trinucleotide repeat sequence [1]. Symptoms include involuntary movements, behavioral problems, and cognitive impairments that typically begin at age 40 [2,3]. For about 5–6% of HD patients, symptoms will develop by age 20, which is called Juvenile-onset HD (JoHD) [4,5]. Motor symptoms of JoHD patients tend to be more rigid and bradykinetic than their adult-onset counterparts [6].

Brain imaging data in JoHD is limited and our group has a unique prospective study evaluating children and young adults. We’ve previously shown that early in the course of disease, the major area of atrophy is specifically the subcortical structures with cortical volumes being relatively spared [7,8]. Functional connectivity from resting state MRI allows evaluation of the integrity of neural circuitry, allowing for detection of more subtle abnormalities despite relatively normal volumes of tissue.

The current study investigated group differences in canonical cortical resting-state functional connectivity networks and subcortical connectivity between JoHD and control participants and whether functional, motor, and cognitive outcome measures related to these differences. Given the relative sparing of cortical volume early in the course of the disease, we hypothesized that there may not be widespread connectivity aberration, however striatal connectivity would be significantly altered.

## 2. Materials and Methods

### 2.1. Sample

Sample selection for this study was conducted utilizing previous work from this lab [8]. Participants for the JoHD group were part of the University of Iowa’s Kids-JoHD study, which enrolled participants between 5 and 26 years old with a clinician-confirmed JoHD diagnosis and accompanying CAG repeat expansion from across the United States between August 2012 and February 2020. Potential subjects were excluded if they indicated a history of head trauma or brain surgery. While the study was longitudinal, only the first visit with resting-state functional MRI scans was used for this project. A comparison group who participated in the University of Iowa’s Kids-HD study was comprised of children at risk for HD due to an HD-diagnosed parent or grandparent. Children underwent genetic testing to ascertain their CAG repeat length, and only participants having fewer than 36 CAG repeats (gene-non-expanded; GNE) were included in the control group. Importantly, this genetic testing occurred for research purposes only, and the results were not shared with the participants, their family, or the study personnel directly interacting with them. A single lab member with no participant and family interactions deidentified all genetic and clinical assessments before data processing and analysis could begin. These procedures were approved by the Institutional Review Board with participant safety and research ethics in mind. Because JoHD participants previously exhibited symptoms and completed genetic testing to determine their clinical diagnosis, their genetic status was known to both the subject and their family.

### 2.2. Magnetic Resonance Imaging

Most MRI scans (51 control and 11 JoHD participants) were completed on 3T Siemens Trio TIM (Siemens AG, Munich, Germany) prior to a scanner replacement in 2016. Later scans (13 control and 8 JoHD) were performed using a 3T General Electric Discovery MR750w (GE Medical Systems, Chicago, IL, USA). Parameters for the T1-weighted anatomical images on the Siemens machine include the following: 1.1 mm isotropic resolution, TR = 2300 ms, TE = 2.87 ms, TI = 900 ms, flip angle = 10°, FOV = 282 × 282 × 264 mm, matrix = 256 × 256 × 240. Resting-state scans were acquired with these parameters: TR = 2000 ms, TE = 3 ms, flip angle = 90°, FOV = 220 × 220 mm, matrix = 64 × 64, slice thickness = 3.5 mm. The GE scanner had similar parameters: 1.1 mm isotropic resolution, TR = 8.392 ms, TE = 3.1 ms, TI = 900 ms, flip angle = 12°, FOV = 282 × 282 × 260 mm, matrix = 256 × 256 × 236. In addition, images collected on the GE scanner utilized real-time prospective motion correction to reduce motion-related artifacts [9]. Resting-state images were collected with the following parameters: TR = 2500 ms, TE = 3 ms, flip angle = 80°, FOV = 220 × 220 mm, matrix = 64 × 64, slice thickness = 3.6 mm.

Anatomical images were corrected for scanner noise and intensity inhomogeneity using the Rician denoising and N4 algorithm, respectively, from Advanced Normalization Tools (ANTs), followed by rigid alignment to the HCPICBM template [10]. Brains were extracted and mask were created before the images were normalized to the HCPICBM template. Resting-state BOLD scans were corrected for motion and registered to template space. Motion regressors were extracted, and their temporal derivatives were calculated. Spatial regressors for signal from cerebrospinal fluid and white matter were obtained using aCompcorr [11]. Nuisance regression of these values, including a threshold for scrubbing of 0.5mm framewise displacement (FD), with a bandpass filter between 0.01 and 0.08 were completed. Because subjects are young with a rare neurodegenerative movement-related disorder, this more liberal motion threshold was utilized. A Welch Two Sample *t*-test was conducted to assess group differences in motion. The average FD did not differ between groups (controls = 0.20, JoHD = 0.32; *t*(18.90) = −1.70, *p* = 0.11. Using the 100-network parcellation identified by Schaefer et al. [12], averaged BOLD time series were extracted for each parcel and correlated with time series from other parcels from the same network and hemisphere. For example, the left visual network connectivity (i.e., within network connectivity) was calculated by correlating each parcel attributed to the left visual network (labels 1–9 of the Schaefer 100-parcellation 7-network atlas) with other left visual network parcels. These 36 values were averaged to obtain an average within-network connectivity value for the left Visual Network, which represents how well regions within each network communicate with other same-network regions. This process was repeated with the remaining 13 bilateral networks: labels 10–15 for left Somatomotor Network, 16–23 for left Dorsal Attention Network (DAN), 24–30 for left Salience Network, 31–33 for left Limbic Network, 34–37 for left Control Network, and 38–50 for left Default Mode Network (DMN). The right hemisphere was completed in the same manner: labels 51–58 for the right Visual Network, 59–66 for the right Somatomotor Network, 67–73 for right DAN, 74–78 for right Salience Network, 79–80 for right Limbic Network, 81–89 for right Control Network, and 90–100 for the right DMN. All values were Fisher-transformed for analysis. In addition to the canonical cortical networks, the left and right caudate and putamen were obtained from the atlas used for BrainsTOOLs (Version v5.4.0. Manufacturer: BRAINSia, University of Iowa, Iowa City, IA, USA) [13], a well-validated software package for analyzing anatomical brain differences. The appropriate label values were selected, binarized, and combined (as needed) using fslmaths [14] to create nine striatal ROI masks that were defined as left, right, and bilateral (combined masks of left and right side) regions of the striatum (combined masks of caudate and putamen), caudate, and putamen. Lastly, the average BOLD time series was extracted for the nine striatal ROIs. To mitigate risks of a type II error, the striatal connectivity analysis was limited to evaluating connections between the striatum and only those canonical cortical circuits that showed significant group difference.

### 2.3. Measures

Disease burden was calculated by multiplying the participant age at their visit by the number of CAG repeats, with higher scores indicating higher disease burden. Thirty-one motor tasks from the Unified Huntington’s Disease Rating Scale (UHDRS) provided a total motor score (TMS) [15]. Total scores from the Schultz Scale [8], a UHDRS subscale comprised of ten motor tasks (dysarthria, left and right upper extremity dystonia, gait, tandem walking, left- and right-hand pronate/supinate, left- and right-hand finger tapping, and tongue protrusion) that best correspond to changes observed in JoHD disease progression, were also utilized as a motor outcome measure. An overall general ability index (GAI) was computed using the Weschler Intelligence Scale for Children aged 5 to 16 [16] and the Weschler Adult Intelligence Scale [17] for those over 16 years old.

### 2.4. Statistical Analyses

Pearson correlations between each network’s average BOLD signal were calculated, transformed to Fisher Z scores, and added to linear mixed-effects (LME) models to determine group differences in connectivity. Because no current studies have analyzed canonical resting-state functional connectivity networks, a simple model examined HD group differences, along with fixed effects of age and sex with a random effect for scanner model. Results were adjusted for multiple comparison using Benjamini & Hochberg’s FDR correction [18]. Using the same model, connectivity between significant networks and striatal ROIs, as well as within-striatal connectivity, was compared.

To ascertain whether network connectivity predicted clinical, motor, and cognitive measures, LME models for each outcome measure (TMS, Schultz Scale total score, disease burden, and GAI) were predicted by connectivity of networks with significant group findings and the fixed effects of age and sex, with a random effect for scanner.

Additional analyses were also conducted to examine scanner and nonlinear age effects associated with cortical networks. Details are reported in the Section 3 and Supplementary Materials.

## 3. Results

### 3.1. Participant Demographics

There were 64 control subjects and 19 with JoHD. While the JoHD group was slightly older (17.35 vs. 14.33 years old) and had more CAG repeats (66.84 vs. 30.31) than the control group, there were no group differences in sex. Disease duration in the JoHD group averaged 4.08 years with diagnosis occurring when they were 13.67 years old (Table 1)

### 3.2. Cortical Network Group Differences

**Figure 1:**
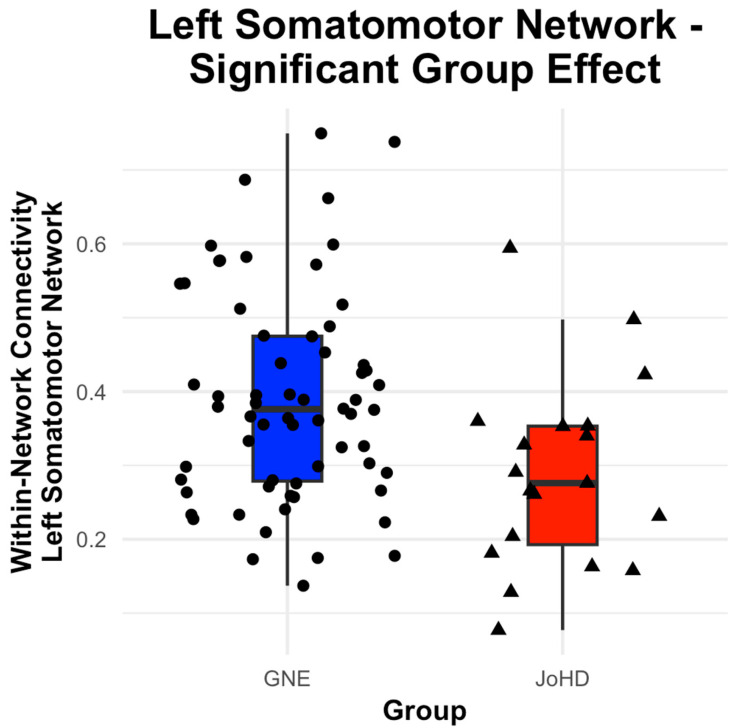
JoHD subjects revealed significantly decreased left Somatomotor Network connectivity compared to control subjects. Within-Network connectivity in the left Somatomotor Network was calculated as the averaged network connectivity between all left somatomotor parcels in the Schaefer 7-network, 100-parcellation atlas. JoHD: Juvenile-Onset Huntington’s Disease.

**Figure 2:**
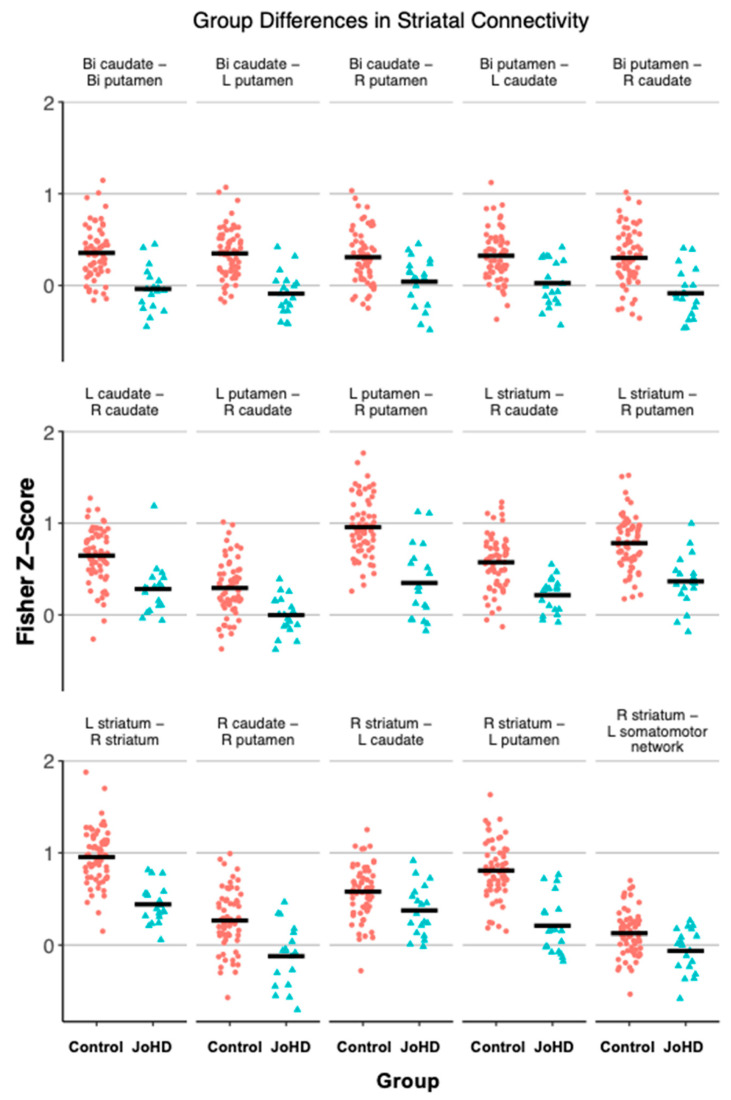
Striatal hypoconnectivity in the JoHD group vs. control (GNE) group is shown here. L = Left, R = Right, Bi = Bilateral.

The left Somatomotor Network connectivity was significantly decreased in the JoHD group compared to the control group, F(1, 78.23) = 6.21, *p*-adjusted = 0.03 (Figure 1). Further, among all participants, left Somatomotor Network was higher in younger vs. older subjects, F(1, 78.97) = 4.67, *p*-adjusted = 0.04. No other network models were significant (all *p*-adjusted > 0.05) (Figure 2 and Table 2).

### 3.3. Striatal Group Differences

Reduced within-striatal connectivity was repeatedly found in the JoHD group compared to the control group (Table 3, Figure 2). Left, right, and bilateral putamen, caudate, and striatal ROIs were hypo-connected to their contralateral and ipsilateral counterparts apart from the left caudate to both the right and left putamen. Further, the left somatomotor network and right striatum displayed reduced connectivity. No other somatomotor network to striatal ROIs were significant (all *p*-adjusted > 0.05).

### 3.4. Outcome Measures

**Figure 3:**
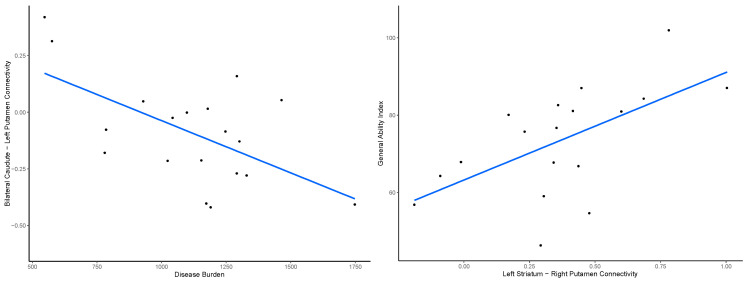
Disease burden among JoHD patients was inversely correlated with bilateral caudate to left putamen connectivity, while increased connectivity between the left striatum and right putamen predicted higher GAI (general ability index).

Motor, functional, and cognitive measures were not significantly related to left somatomotor network connectivity (all *p*’s > 0.05). Within the striatum, connectivity between the left striatum and right putamen was positively related to GAI, *t*(14) = 3.35, *p*-adjusted = 0.009. Disease burden inversely predicted bilateral caudate to left putamen connectivity (*t*(16) = −3.04, *p*-adjusted = 0.008). Participants with increased connectivity between these striatal regions scored higher in general ability index and had lower disease burden (Figure 3).

### 3.5. Scanner and Nonlinear Age Effects

Given the developmental complexity of adolescent functional connectivity, we explored potential nonlinear effects of age by adding an age^2^ term to the cortical network models, resulting in models with main effects for group, linear age, nonlinear age, sex, and a random effect of scanner model. In the left Somatomotor Network, the main effect of group remained significant, while both linear and nonlinear age terms were nonsignificant. Only the left Salience Network showed significant linear and nonlinear age effects (adjusted p’s = 0.005 and 0.006, respectively). Plots and expanded discussion of these age-related trends are provided in Supplementary Materials (Figures S1 and S2, Table S1).

To evaluate the impact of using two MRI scanners, group by scanner interaction terms were added to the connectivity models, and the random effect was removed. The main effect of group remained unchanged: the left Somatomotor Network continued to show a significant effect. Scanner model was a significant predictor for left Dorsal Attention, left Control, right Somatomotor, and right Dorsal Attention networks, with greater connectivity detected on the Siemens scanner. These results are detailed in Supplementary Materials (Table S2, Figure S3).

## 4. Discussion

The current analysis demonstrated typical within-network functional connectivity in the cortex among JoHD patients, with the exception of reduced connectivity in the left somatomotor network. Though significantly different than the control group, left somatomotor connectivity was not related to common JoHD clinical, motor, or cognitive measures. Hypoconnectivity within the striatum, as well as from the right striatum to left somatomotor network was displayed in the JoHD group. Disease burden inversely predicted bilateral caudate–left putamen connectivity, while left striatum–right putamen connectivity positively corelated to general ability index scores.

These findings are in line with our previous volumetric analysis of early-course JoHD (on average, within 4 years of diagnosis), in which the striatum is severely atrophied while the cerebral cortex is relatively spared in volume [8,19]. In our most recent analysis of children at risk for HD, we have found that as far back as 50 years from onset, children with the HD gene expansion (gene-expanded or GE group) have an enlarged cortex and higher IQ compared to children at risk for HD but who did not inherit the expanded allele (gene non-expanded or GNE group) [20]. This is conceptualized as the HD gene having antagonistic pleiotropy properties in which early advantage is paired with later liability for degeneration. It is also hypothesized that the enlarged cortex provides excessive glutamatergic input to the striatum that later in life gives way to excitotoxicity and neurodegeneration [21]. In the [20] study, there were no children with CAG repeats greater than 60, and no children who currently had symptoms were included. However, 50 years prior to HD onset, children in the GE group had nearly 40 ml more cortical gray matter than those in the GNE group. Over the next several decades, this volume declined linearly, such that by 10 years prior to onset, the GE group had approximately 30 ml less cortical gray matter than the GNE group. Therefore, there is no direct evidence that there is enlarged cortex early in life in those with JoHD. However, given our previous analysis of JoHD in which the cortex was relatively spared, it makes intuitive sense that the same process of early cortical enlargement is also occurring, even with very large repeats.

In the current study, the only abnormal cortical circuitry was the left somatomotor circuit, showing both within-network lower connectivity as well as reduced connectivity to the striatum. These findings can also be construed as support for antagonistic pleiotropy with cells and circuits displaying early high functioning are exactly those same cells and circuits that are vulnerable to degeneration. For example, [22] showed that areas of the cortex with high expression of developmental genes were the same areas that later had degeneration in HD. Also, a recent study of isogenic human embryonic cortical stem cells found that expanded CAG repeats let to accelerated neuronal maturation and longer neurites, suggestive of superior function. Yet, these same neurons died significantly earlier, highlighting the trade-off between advantageous development and early degeneration [23]. Our findings also support the glutamate toxicity hypothesis as the regions within the somatomotor network are the ones with the highest glutamatergic connections to the striatum [24]. Finally, the current findings of abnormalities in the cortical circuits were lateralized to the left somatomotor circuit. A recent study using post-mortem MRIs showed the atrophy in HD to have a significant laterality with the left hemisphere preferentially affected by degeneration compared to the right [25]. The underlying mechanism for left-sided vulnerability in HD could be associated with several co-occurring processes. Higher rates of right-handedness could lead to a more functionally refined left hemisphere, including a robust glutamatergic somatomotor-striatal pathway. These neurons are susceptible to early degeneration, leading to structural atrophy and reduced connectivity, as shown in this study.

Because connectivity between the left striatum and right putamen predicts higher GAI, this circuit may serve as a biomarker for preserved cognitive function. Early intervention trials for HD often focus on delaying motor onset via gene slicing or editing [26]. A complementary therapeutic approach could focus on maintaining striatal integrity to mitigate cognitive declines. Structural and functional imaging provide a non-invasive, repeatable measure with established processing pipelines for longitudinal striatal changes.

There are several strengths and weaknesses in this study. JoHD is severely understudied due to disease rarity, yet the project here provides a wealth of data in a relatively large group of subjects. This project is the first to look at within-network connectivity in JoHD patients and provides further evidence of early life compensation prior to disease onset. However, more pre-adolescent JoHD participants would allow for further age investigations, as well as increased power to see significantly different clinical and motor outcomes. Although both groups included children as young as six years old, the JoHD group primarily ranged from 15 to 25 years, whereas the control group spanned from 6 to 20 years. This pattern reflects several important clinical and logistical realities. First, earlier motor onset in JoHD is typically associated with extremely high CAG repeat lengths, which are exceptionally rare [6]. As a result, JoHD cases with motor onset before age 10 are uncommon. Young children may experience delays in diagnosis due to required visible motor symptoms, clinician confirmation, and genetic testing for confirmation [27]. Children with early-onset JoHD often further exhibit developmental, behavioral, or cognitive impairments that may limit their ability to participate in structured research protocols [6,28]. Logistical barriers also play a significant role. Parents of JoHD children may face time constraints, limited flexibility, or added caregiving responsibilities — especially if they are HD gene carriers themselves or caring for other affected family members. To mitigate these challenges in future research, studies could consider coordinating visits with routine clinical appointments, offering caregiver support services, or incorporating remote or in-home assessments to reduce burden on families. In addition, investigating longitudinal changes in connectivity could help elucidate the trajectory differences in JoHD compared to adult-onset HD. Lastly, resting-state fMRI does not directly measure neuronal activity, which limits the extent to which testing can confirm underlying cellular mechanisms. Future studies should incorporate multimodal approaches test hypotheses with greater specificity and confidence. For example, combining fMRI with MR spectroscopy would allow future researchers to quantify the relationship between glutamate levels and striatal connectivity. Animal models using optogenetics alongside fMRI could further clarify how targeted neural manipulations influence network-level functions. Given the rarity of this disease, multimodal designs may offer a more efficient and informative use of limited participant data.

## 5. Conclusions

In summary, this study provided further information regarding functional connectivity in the JoHD brain. Recapitulating the findings in our volumetric studies, the only canonical cortical network abnormality was in the left somatomotor cortex, and its connections to the striatum. These findings lend further support to theories of antagonistic pleiotropy with early high-functioning circuits leading to later early degeneration of those same circuits, potentially due to glutamate excitotoxicity. Finally, the findings add to the recent evidence of the degeneration of the process being left-lateralized.

## Figures and Tables

**Table 1:** Sample Demographics.

	Control (*n* = 64)	JoHD (*n* = 19)		
	*n* (%)	*n* (%)	*X* ^2^	*p*
Age (mean ± standard deviation)	14.33 ± 3.25	17.35 ± 5.75	*t* = −2.19	0.04
Sex			*X*^2^ = 0	1
Female	38 (59%)	11 (58%)		
Male	26 (41%)	8 (42%)		
CAG Repeats	20.31 ± 4.16	66.84 ± 10.97	−18.11	<0.001
Age at Diagnosis	NA	13.67 ± 4.86	NA	NA
Disease Duration	NA	4.08 ± 2.92	NA	NA

JoHD: Juvenile-Onset Huntington’s Disease; CAG: Cytosine-Adenine-Guanine repeats in the huntingtin gene; Age at Diagnosis: Age when participant received motor diagnosis based on neurologist’s clinical documentation; Disease Duration: Time elapsed between age at diagnosis and age at study visit.

**Table 2:** Linear Mixed Effects Model Results.

Network	Predictor	Estimate	SE	df	*t*	*p*-adj	95% CI
Left Visual	Group	−0.01	0.03	79.00	−0.41	0.87	[−0.067, 0.043]
	Age	−0.001	0.003	79.00	−0.42	0.87	[−0.007, 0.004]
	Sex	−0.004	0.02	79.00	−0.17	0.87	[−0.049, 0.041]
Left Somatomotor	Group	−0.09	0.04	78.24	−2.50	0.03 *	[−0.163, −0.018]
	Age	−0.01	0.004	78.98	−2.22	0.04 *	[−0.016, 0.000]
	Sex	0.02	0.03	78.06	0.75	0.45	[−0.035, 0.081]
Left Dorsal Attention	Group	0.03	0.04	78.27	0.78	0.58	[−0.052, 0.115]
	Age	−0.01	0.004	78.87	−1.14	0.52	[−0.015, 0.003]
	Sex	−0.01	0.03	78.07	−0.15	0.88	[−0.074, 0.062]
Left Salience	Group	−0.04	0.03	78.34	−1.32	0.25	[−0.090, 0.015]
	Age	0.004	0.003	78.44	1.52	0.25	[−0.002, 0.010]
	Sex	−0.02	0.02	78.09	−0.87	0.39	[−0.062, 0.023]
Left Limbic	Group	0.03	0.06	78.43	0.43	0.67	[−0.091, 0.157]
	Age	−0.01	0.01	77.35	−0.84	0.54	[−0.018, 0.010]
	Sex	−0.08	0.05	78.11	−1.65	0.21	[−0.182, 0.018]
Left Control	Group	0.05	0.04	78.33	1.35	0.28	[−0.026, 0.124]
	Age	−0.01	0.004	78.50	−1.26	0.28	[−0.014, 0.002]
	Sex	−0.03	0.03	78.09	−0.88	0.38	[−0.088, 0.032]
Left Default Mode	Group	−0.03	0.02	78.52	−1.42	0.32	[−0.071, 0.009]
	Age	0.0003	0.002	75.51	0.12	0.90	[−0.005, 0.004]
	Sex	−0.01	0.02	78.14	−0.77	0.59	[−0.046, 0.019]
Right Visual	Group	0.02	0.03	78.67	0.55	0.58	[−0.039, 0.078]
	Age	0.003	0.003	70.78	1.00	0.58	[−0.002, 0.010]
	Sex	−0.02	0.02	78.19	−0.71	0.58	[−0.064, 0.031]
Right Somatomotor	Group	0.05	0.04	78.19	1.03	0.41	[−0.042, 0.129]
	Age	−0.01	0.005	78.99	−1.18	0.41	[−0.015, 0.003]
	Sex	0.01	0.04	78.05	0.29	0.77	[−0.060, 0.079]
Right Dorsal Attention	Group	0.02	0.05	78.29	0.47	0.64	[−0.074, 0.114]
	Age	−0.01	0.01	78.79	−0.97	0.64	[−0.016, 0.004]
	Sex	−0.03	0.04	78.07	−0.67	0.64	[−0.103, 0.049]
Right Salience	Group	0.004	0.03	78.26	0.11	0.98	[−0.062, 0.067]
	Age	0.002	0.004	78.93	0.55	0.98	[−0.006, 0.008]
	Sex	0.0006	0.03	78.06	0.02	0.98	[−0.052, 0.052]
Right Limbic	Group	−0.01	0.10	79.00	−0.10	0.92	[−0.194, 0.174]
	Age	−0.01	0.01	79.00	−1.27	0.35	[−0.031, 0.006]
	Sex	0.09	0.08	79.00	1.13	0.35	[−0.063, 0.237]
Right Control	Group	0.02	0.03	78.67	0.67	0.61	[−0.032, 0.073]
	Age	0.002	0.003	70.73	0.54	0.61	[−0.003, 0.008]
	Sex	−0.01	0.02	78.19	−0.51	0.61	[−0.053, 0.032]
Right Default Mode	Group	0.02	0.03	78.67	0.55	0.58	[−0.039, 0.078]
	Age	0.003	0.003	70.78	1.00	0.58	[−0.002, 0.010]
	Sex	−0.02	0.02	78.19	−0.71	0.58	[−0.064, 0.031]

Note. Results of linear mixed effects models reveal group and age differences are significant for left Somatomotor within-network connectivity only. * = significant value.

**Table 3:** Striatal Connectivity Group Results.

Roi Pair	Estimate	SE	df	*t*	*p*-adj	95% CI
Left striatum–right striatum	−0.48	0.08	78.56	−6.21	<0.001 *	[−0.63, −0.33]
Left striatum–right caudate	−0.35	0.08	78.71	−4.64	<0.001 *	[−0.51, −0.21]
Left striatum–right putamen	−0.36	0.08	78.87	−4.61	<0.001 *	[−0.52, −0.21]
Right striatum–left caudate	−0.18	0.08	79	−2.34	0.04 *	[−0.33, −0.03]
Right striatum–left putamen	−0.54	0.08	78.74	−6.59	<0.001 *	[−0.71, −0.39]
Bilateral caudate–bilateral putamen	−0.36	0.07	79	−4.79	<0.001 *	[−0.50, −0.21]
Bilateral caudate–left putamen	−0.40	0.07	79	−5.62	<0.001 *	[−0.53, −0.26]
Bilateral caudate–right putamen	−0.24	0.08	79	−3.04	0.01 *	[−0.39, −0.09]
Left caudate–right caudate	−0.35	0.08	79	−4.36	<0.001 *	[−0.50, −0.19]
Left putamen–right caudate	−0.28	0.08	78.56	−3.69	<0.001 *	[−0.44, −0.14]
Right caudate–right putamen	−0.36	0.09	78.96	−3.98	<0.001 *	[−0.53, −0.19]
Bilateral putamen–right caudate	−0.37	0.08	78.7	−4.37	<0.001 *	[−0.54, −0.21]
Bilateral putamen–left caudate	−0.25	0.07	79	−3.43	<0.001 *	[−0.40, −0.11]
Left putamen–right putamen	−0.50	0.09	78.78	−5.69	<0.001 *	[−0.44, −0.14]
Bilateral striatum–bilateral somatomotor network	−0.13	0.07	79	−1.83	0.20	[−0.68, −0.34]
Left striatum–bilateral somatomotor network	−0.09	0.07	79	−1.15	0.51	[−0.27, 0.01]
Right striatum–bilateral somatomotor network	−0.17	0.07	78.9	−2.51	0.06	[−0.23, 0.06]
Bilateral caudate–bilateral somatomotor network	−0.05	0.06	79	−0.84	0.54	[−0.30, −0.04]
Left caudate–bilateral somatomotor network	0.02	0.07	79	0.25	0.81	[−0.17, 0.07]
Right caudate–bilateral somatomotor network	−0.11	0.06	79	−1.79	0.22	[−0.11, 0.14]
Bilateral putamen–bilateral somatomotor network	−0.14	0.08	78.96	−1.79	0.31	[−0.23, 0.01]
Left putamen–bilateral somatomotor network	−0.14	0.08	79	−1.89	0.15	[−0.29, 0.01]
Right putamen–bilateral somatomotor network	−0.13	0.07	78.62	−1.75	0.34	[−0.29, 0.003]
Bilateral striatum–left somatomotor network	−0.17	0.07	78.96	−2.35	0.09	[−0.28, 0.01]
Left striatum–left somatomotor network	−0.09	0.08	79	−1.14	0.55	[−0.31, −0.03]
Right striatum–left somatomotor network	−0.21	0.07	78.8	−3.17	0.01 *	[−0.24, 0.06]
Bilateral caudate–left somatomotor network	−0.13	0.07	79	−1.78	0.31	[−0.34, −0.08]
Left caudate–left somatomotor network	−0.05	0.07	79	−0.75	0.77	[−0.27, 0.01]
Right caudate–left somatomotor network	−0.15	0.07	79	−2.31	0.09	[−0.20, 0.09]
Bilateral putamen–left somatomotor network	−0.12	0.07	79	−1.69	0.38	[−0.28, −0.03]
Left putamen–left somatomotor network	−0.11	0.08	79	−1.41	0.66	[−0.26, 0.02]
Right putamen–left somatomotor network	−0.12	0.07	79	−1.77	0.33	[−0.26, 0.04]
Bilateral striatum–right somatomotor network	−0.08	0.07	78.51	−1.23	0.61	[−0.26, 0.01]
Left striatum–right somatomotor network	−0.1	0.06	79	−1.61	0.22	[−0.22, 0.04]
Right striatum–right somatomotor network	−0.07	0.07	78.28	−0.98	0.92	[−0.23, 0.02]
Bilateral caudate–right somatomotor network	0.02	0.06	79	0.41	0.68	[−0.20, 0.06]
Left caudate–right somatomotor network	0.04	0.06	78.81	0.64	0.67	[−0.09, 0.13]
Right caudate–right somatomotor network	−0.02	0.06	78.71	−0.3	0.82	[−0.08, 0.16]
Bilateral putamen–right somatomotor network	−0.14	0.08	78.4	−1.8	0.30	[−0.13, 0.09]
Left putamen–right somatomotor network	−0.16	0.08	78.78	−2.07	0.17	[−0.30, 0.01]
Right putamen–right somatomotor network	−0.11	0.07	78.44	−1.46	0.59	[−0.32, −0.02]
Left somatomotor network–right somatomotor network	−0.03	0.09	78.99	−0.34	0.74	[−0.26, 0.03]

Note. Results of Linear Mixed Effects (LME) models revealed striatal hypoconnectivity in JoHD patients compared to controls. * = significant value.

## Data Availability

We are committed to data sharing. The dataset of the present study, including deidentified participant data, processed brain measures, and functional assessments, will be made available upon reasonable request. Requests may be made to the corresponding authors, and approval from the sponsoring institution (University of Iowa) is required.

## Supplementary Materials

The following supporting information can be downloaded at: https://www.mdpi.com/article/10.3390/brainsci15060663/s1, Figure S1: Nonlinear Age Effects by Group for All Networks; Figure S2: Linear Age Effects by Group for All Networks; Figure S3: Scanner Differences Across Network; Table S1: Nonlinear Age Models; Table S2: Scanner by Group Interaction Models.

## Abbreviations

The following abbreviations are used in this manuscript:

JoHD	Juvenile-onset Huntington’s Disease
HD	Huntington’s Disease
GE	Gene-expanded
GNE	Gene-non-expanded
CAG	Cytosine-adenine-guanine
MRI	Magnetic resonance imaging
GAI	General ability index
ROI	Region of interest
UHDRS	Unified Huntington’s Disease Rating Scale
TMS	Total motor score
LME	Linear Mixed-Effects
