# Supplementary material for: Cortical and Striatal Functional Connectivity in Juvenile-Onset Huntington’s Disease
Source: Brain Sci. 2025 Jun 19;15(6):663. doi: 10.3390/brainsci15060663 (PMC12191134; doi:10.3390/brainsci15060663)
Supplement: Supplementary file 1 [file brainsci-15-00663-s001.zip › brainsci-3672398-supplementary.pdf]

Supplementary Materials

Linear and Nonlinear Age Effects

We examined whether age-related changes in functional connectivity followed a curvilinear pattern. Age^2 was added to the original models to test for nonlinear effects. In the model for the left Somatomotor Network, the main effect of group remained significant, but neither the linear nor nonlinear age terms were significant. Notably, linear age effects became nonsignificant once the nonlinear term was included, suggesting that developmental changes in connectivity may follow a curvilinear rather than linear trajectory in this network. The scatterplot of age and connectivity shows greater variability before age 20, implying that developmental shifts may be most pronounced in early adolescence.

Of the other 13 networks, only the left Salience Network demonstrated significant linear and nonlinear age effects ($F(1, 77.34) = 11.56$, p-adjusted = 0.01; $F(1, 77.80) = 9.85$, p-adjusted = 0.01). This pattern followed an inverted U-shaped curve, with connectivity peaking in mid-adolescence. This may reflect heightened salience network activity during a developmental period when rapid evaluation of emotional and environmental stimuli is particularly adaptive.

Figure S1. Nonlinear Age Effects by Group for All Networks

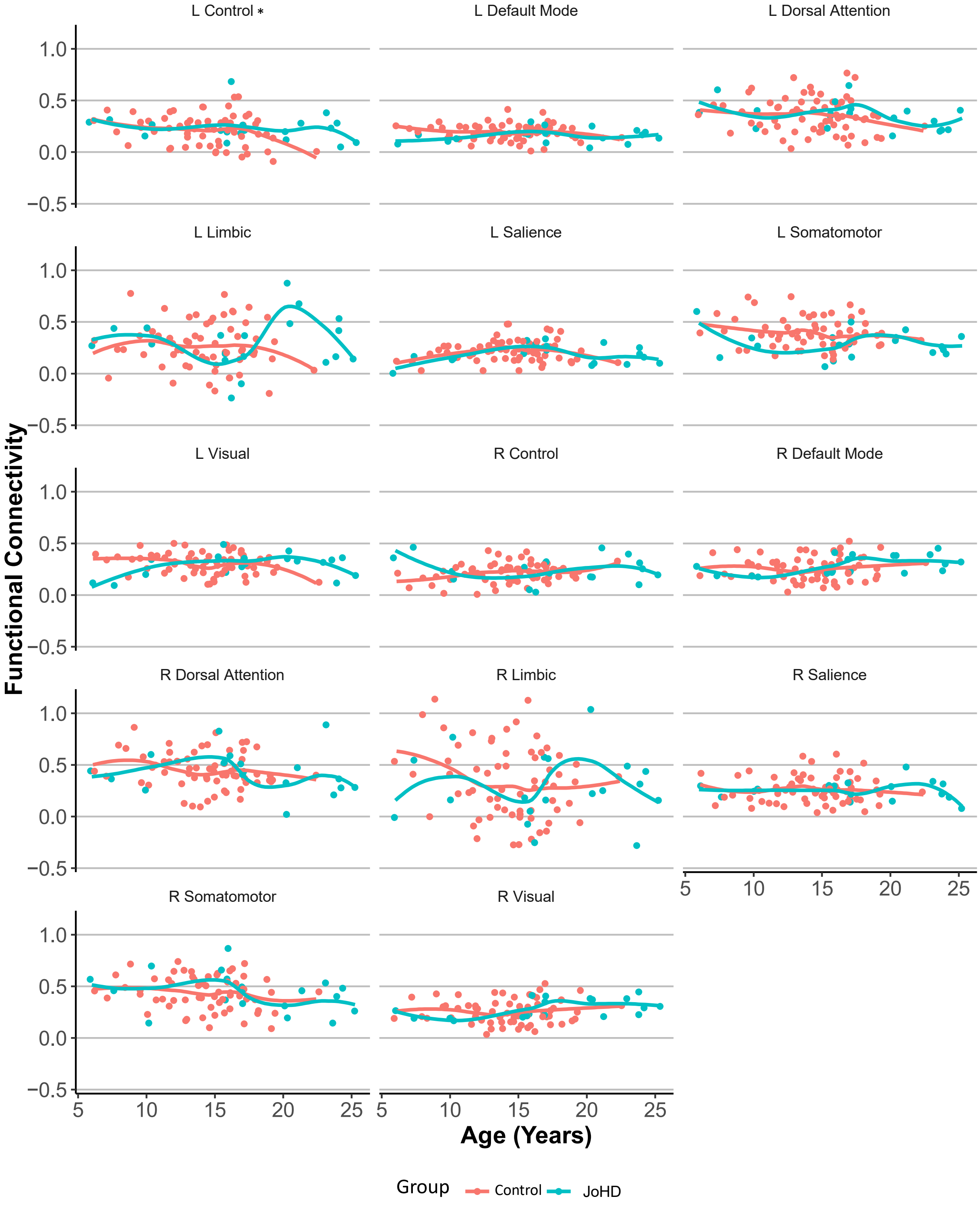

Note. Networks with * are significant

Figure S2: Linear Age Effects by Group for All Networks

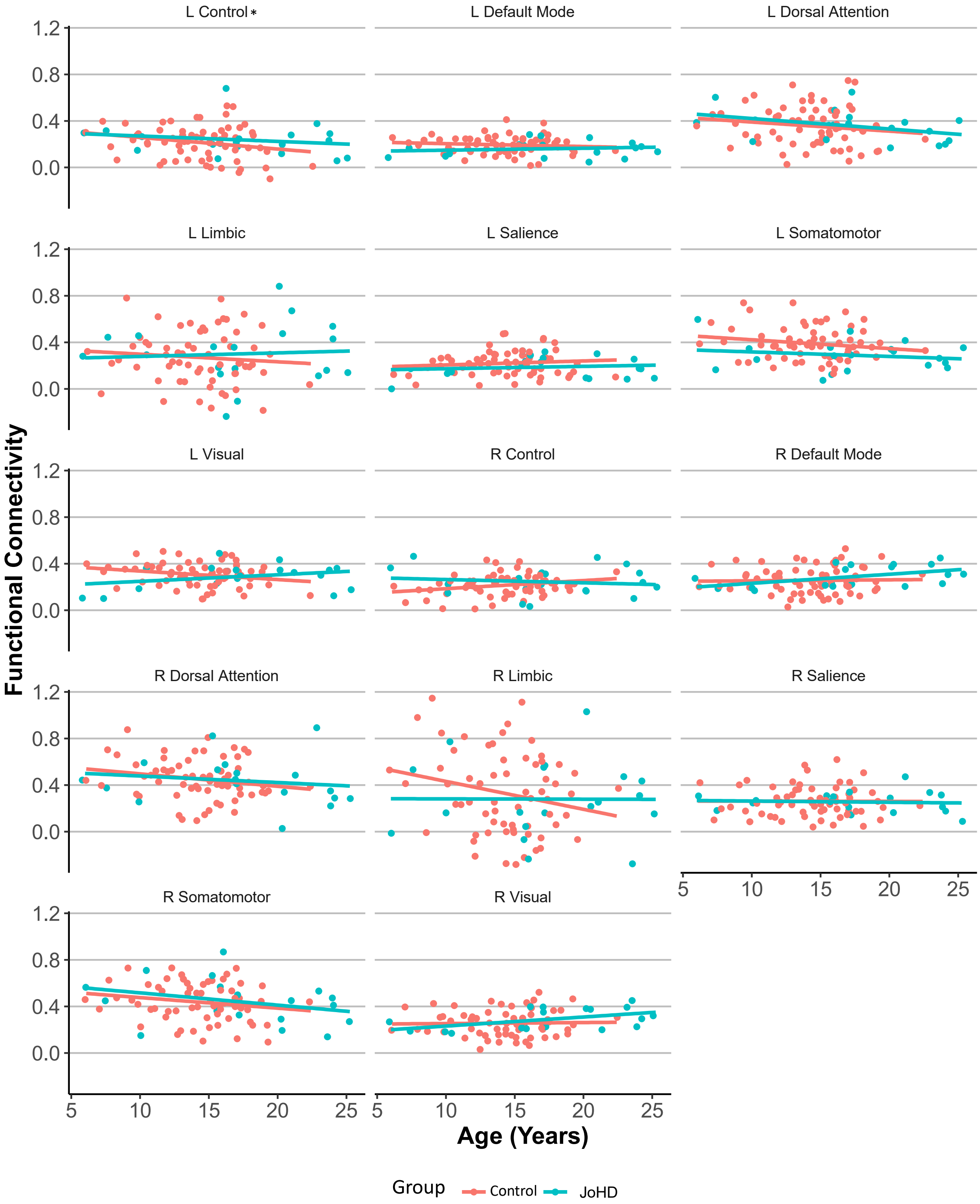

Note. Networks with * are significant

Table S1: Nonlinear Age Models

Network	Predictor	F	df	df residual	p-adj
Left Visual	Group	0.03	1	77.13	0.99
	Age	1.21	1	77.87	0.46
	Age^2	1.34	1	77.15	0.46
	Sex	0.00009	1	77.14	0.99
Left Somatomotor	Group	6.32	1	77.02	0.04*
	Age	1.09	1	77.18	0.50
	Age^2	0.40	1	77.48	0.53
	Sex	0.43	1	77.02	0.53
Left Dorsal Attention	Group	0.84	1	77.02	0.91
	Age	0.07	1	77.20	0.94
	Age^2	0.24	1	77.54	0.94
	Sex	0.01	1	77.02	0.94
Left Salience	Group	0.02	1	77.04	0.90
	Age	11.56	1	77.34	0.01*
	Age^2	9.85	1	77.80	0.01*
	Sex	0.22	1	77.04	0.80
Left Limbic	Group	0.04	1	77.04	0.85
	Age	0.36	1	77.33	0.82
	Age^2	0.20	1	77.79	0.82
	Sex	2.83	1	77.04	0.24
Left Control	Group	1.57	1	77.03	0.54
	Age	0.01	1	77.24	0.91
	Age^2	0.01	1	77.62	0.91
	Sex	0.71	1	77.03	0.67
Left Default Mode	Group	1.49	1	77.04	0.57
	Age	0.02	1	77.39	0.91
	Age^2	0.01	1	77.88	0.91
	Sex	0.56	1	77.05	0.76
Right Visual	Group	0.01	1	77.07	0.94
	Age	0.57	1	77.58	0.57
	Age^2	0.90	1	78.00	0.57
	Sex	0.69	1	77.08	0.57
Right Somatomotor	Group	1.37	1	77.02	0.61
	Age	0.11	1	77.14	0.74
	Age^2	0.31	1	77.39	0.74
	Sex	0.13	1	77.02	0.74
Right Dorsal Attention	Group	0.20	1	77.02	0.95
	Age	0.01	1	77.21	0.95
	Age^2	0.004	1	77.55	0.95
	Sex	0.42	1	77.03	0.95
Right Salience	Group	0.08	1	77.02	0.94
	Age	0.27	1	77.19	0.94

	Age^2	0.18	1	77.51	0.94
	Sex	0.01	1	77.02	0.94
Right Limbic	Group	0.28	1	77.13	0.60
	Age	1.37	1	77.87	0.44
	Age^2	0.87	1	77.15	0.44
	Sex	0.94	1	77.14	0.44
Right Control	Group	0.26	1	77.06	0.92
	Age	0.01	1	77.51	0.92
	Age^2	0.04	1	77.99	0.92
	Sex	0.28	1	77.07	0.92
Right Default Mode	Group	0.01	1	77.07	0.94
	Age	0.57	1	77.58	0.57
	Age^2	0.90	1	78.00	0.57
	Sex	0.69	1	77.08	0.57

Note. * = significant

Scanner Differences

Scanner Model Considerations and Group by Scanner Analyses

In response to our institution installing a new scanner, several steps were taken to evaluate and mitigate potential confounding effects related to our mid-study scanner upgrade. Although the Siemens Prisma scanner offers improved hardware and signal quality, we recognize that its use alongside the GE scanner could introduce unwanted variance.

1. Protocol Harmonization: During the transition, we adjusted MRI acquisition parameters to minimize differences in spatial and temporal resolution (e.g., maintaining comparable TR/TE).
2. Motion Comparison: A comparison of framewise displacement (FD) between scanners revealed no significant differences, $t(54.17) = 0.90$, $p = 0.37$, suggesting that motion artifacts were comparable across platforms despite GE's real-time prospective motion correction.
3. Group-by-Scanner Distribution: A chi-square test found no significant association between scanner type and group membership (Control vs JoHD), $\chi^2(1) = 2.50$, $p = 0.11$.
4. Group by Scanner Interaction: To directly address scanner-related confounds, all functional connectivity models were re-run including a group × scanner interaction term. No changes emerged in the main effect of group. The left Somatomotor Network remained significant. However, scanner model showed significant main effects in four networks: left Dorsal Attention, left Control, right Somatomotor, and right Dorsal Attention, with greater connectivity observed in participants scanned on the Siemens scanner.

The anatomical overlap of these networks — primarily in dorsal and lateral parietal and posterior frontal regions — suggests potential susceptibility to scanner-specific differences, possibly due to field inhomogeneities, gradient nonlinearity corrections, or differences in RF coil design. Although no group by scanner interactions were significant, we recommend thoughtful interpretation, nonetheless.

Figure S3. Scanner Differences Across Network

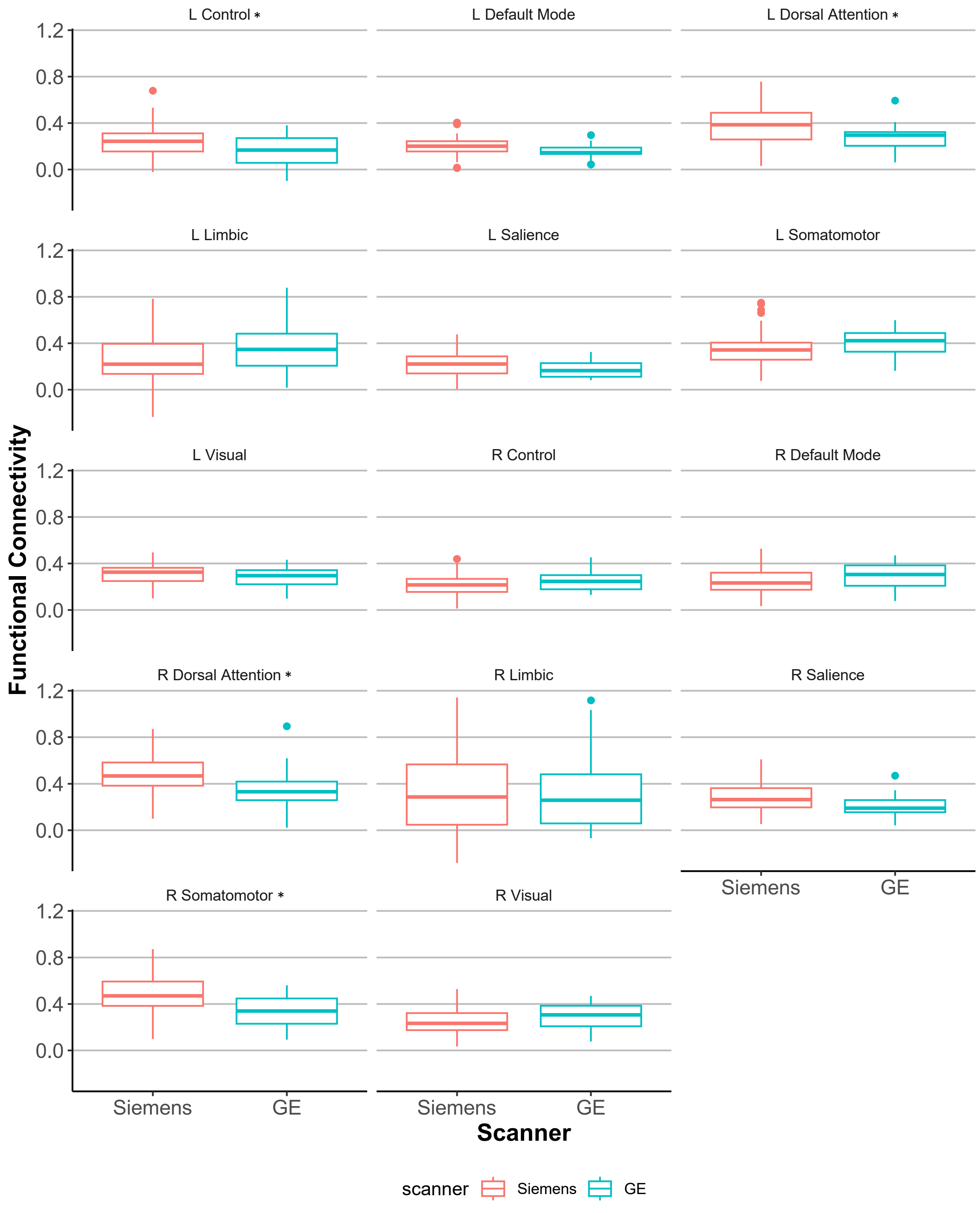

Note. Networks with * are significant

Table S2: Scanner by Group Interaction Models

Network	Variable	F	df	df residual	p-adj
Left Visual	Group	0.31	1	77	0.93
	Scanner	0.97	1	77	0.93
	Age	0.01	1	77	0.93
	Sex	0.01	1	77	0.93
	Group x Scanner	0.16	1	77	0.93
Left Somatomotor	Group	8.28	1	77	0.03*
	Scanner	4.49	1	77	0.06
	Age	5.64	1	77	0.05**
	Sex	0.54	1	77	0.46
	Group x Scanner	1.15	1	77	0.36
Left Dorsal Attention	Group	0.002	1	77	0.96
	Scanner	9.49	1	77	0.01*
	Age	1.02	1	77	0.53
	Sex	0.02	1	77	0.96
	Group x Scanner	3.73	1	77	0.14
Left Salience	Group	1.61	1	77	0.35
	Scanner	3.30	1	77	0.24
	Age	2.85	1	77	0.24
	Sex	0.70	1	77	0.51
	Group x Scanner	0.22	1	77	0.64
Left Limbic	Group	0.27	1	77	0.60
	Scanner	2.78	1	77	0.25
	Age	0.83	1	77	0.46
	Sex	2.81	1	77	0.25
	Group x Scanner	1.30	1	77	0.43
Left Control	Group	0.35	1	77	0.56
	Scanner	7.87	1	77	0.03*
	Age	1.11	1	77	0.50
	Sex	0.73	1	77	0.50
	Group x Scanner	1.08	1	77	0.50
Left Default Mode	Group	2.93	1	77	0.23
	Scanner	3.09	1	77	0.23
	Age	0.12	1	77	0.73
	Sex	0.55	1	77	0.73
	Group x Scanner	0.21	1	77	0.73
Right Visual	Group	1.25	1	77	0.46
	Scanner	3.28	1	77	0.37
	Age	0.63	1	77	0.46
	Sex	0.56	1	77	0.46
	Group x Scanner	0.81	1	77	0.46
Right Somatomotor	Group	0.004	1	77	0.95
	Scanner	12.78	1	77	0.003*
	Age	1.17	1	77	0.71

	Sex	0.09	1	77	0.95
	Group x Scanner	0.56	1	77	0.76
	Group	0.08	1	77	0.77
	Scanner	8.39	1	77	0.02*
Right Dorsal Attention	Age	0.63	1	77	0.65
	Sex	0.42	1	77	0.65
	Group x Scanner	0.94	1	77	0.65
	Group	0.04	1	77	0.96
	Scanner	6.45	1	77	0.07
Right Salience	Age	0.45	1	77	0.84
	Sex	0.002	1	77	0.96
	Group x Scanner	1.45	1	77	0.58
	Group	0.29	1	77	0.74
	Scanner	0.08	1	77	0.78
Right Limbic	Age	2.22	1	77	0.47
	Sex	1.18	1	77	0.47
	Group x Scanner	1.60	1	77	0.47
	Group	1.16	1	77	0.71
	Scanner	2.75	1	77	0.51
Right Control	Age	0.11	1	77	0.74
	Sex	0.30	1	77	0.73
	Group x Scanner	0.44	1	77	0.73
	Group	1.25	1	77	0.46
	Scanner	3.28	1	77	0.37
Right Default Mode	Age	0.63	1	77	0.46
	Sex	0.56	1	77	0.46
	Group x Scanner	0.81	1	77	0.46

Note. * = significant. ** = p-value rounded down to 0.05.
